# Environmental and Phylogenetic Investigations of *Aspergillus flavus* Outbreak Linked to Contaminated Building Materials, Denmark, 2025

**DOI:** 10.3201/eid3203.251219

**Published:** 2026-03

**Authors:** Alexander Gewecke, Raphael Niklaus Sieber, Søren Hallstrøm, Marc Stegger, Barbara Kolarik, Jenny D. Knudsen, Birgitte Andersen, Astrid Hall, Nadja Hawwa Vissing, Maiken Cavling Arendrup

**Affiliations:** Statens Serum Institut, Copenhagen, Denmark (A. Gewecke, R.N. Sieber, S. Hallstrøm, M. Stegger, M.C. Arendrup); Harry Butler Institute, Murdoch University, Perth, Western Australia, Australia (M. Stegger); dansk MiljøAnalyse, Vedbæk, Denmark (B. Kolarik); Copenhagen University Hospital, Rigshospitalet, Copenhagen (J.D. Knudsen, A. Hall, N.H. Vissing, M.C. Arendrup); Aalborg University, Copenhagen (B. Andersen); University of Copenhagen, Copenhagen (M.C. Arendrup)

**Keywords:** Aspergillus flavus, fungi, aspergillosis, environmental sampling, environmental monitoring, air microbiology, surface microbiology, whole-genome sequencing, genotyping techniques, phylogeny, molecular epidemiology, hospital outbreak/nosocomial outbreak, environmental contamination, fungal burden, building materials, clonal expansion, outbreak, outbreak source, Denmark

## Abstract

An *Aspergillus flavus* outbreak occurred in a tertiary hospital in Denmark. We compared environmental sampling methods, investigated the outbreak through short tandem-repeat genotyping, STR*Afla*, and analyzed isolate phylogeny using whole-genome sequencing. Paired sampling revealed that air sampling underestimated *A. flavus* burden (8 CFU/81 air samples vs. 585 CFU/81 surface samples), and culturing at 37°C was superior to 25°C (risk ratio 1.77; p<0.001). STR*Afla* (n = 145) confirmed clonality of the outbreak isolates. Active growth was identified in a kitchen inside the affected ward. Genetically related isolates were also found in the Department of Clinical Microbiology and in 4 unrelated wood-based building materials from retailers in Denmark. Phylogenetic analyses of 167 isolates supported introduction of *A. flavus* from building materials. We hypothesize that water damage enabled germination of dormant spores in precontaminated wood-based products. Our findings highlight a risk factor for outbreaks and should inform future hospital construction and infection prevention strategies.

*Aspergillus flavus,* the second leading cause of invasive aspergillosis (IA) worldwide, is an opportunistic mold that can cause severe infection, including IA, in immunocompromised patients (*1*). It predominates in Asia, the Middle East, and Africa because of its resilience in arid climates; however, because of climate change, its presence in the Northern hemisphere is expected to increase (*1*; N. van Rhijn, unpub. data, https://www.researchsquare.com/article/rs-6545782/v1). Hospital outbreaks mainly occur when airborne conidia are dispersed during construction work (*2*). The lower respiratory tract is usually the affected site of infection; in hematology patients, fatality rates are often high (*2*). 

Renovations during 2017–2019 in a pediatric hematology ward at the largest tertiary hospital in Denmark likely led to infections in 6 patients by spreading conidia (*3*). Infections occurred despite amphotericin B prophylaxis, consistent with the intrinsic reduced susceptibility of *A. flavus*. Transition to posaconazole prophylaxis was implemented and prevented *A. flavus* in patients with adequate serum drug levels. In 2022–2023, we examined the genetic epidemiology of the outbreak using short tandem repeat (STR) genotyping, STR*Afla* (*4*,*5*). We confirmed isogeneity and linked 12 additional patients to the outbreak. Analysis of air samples also indicated the presence of *A. flavus* outbreak isolates in the ward, suggesting its undetected perseverance in the hospital over time. However, 8 more patients positive for outbreak-related *A. flavus* appeared during April 2023–January 2024; of those, 3 were from the outbreak ward floor and 5 were from other parts of the hospital, including a staff member in the Department of Clinical Microbiology (DCM). 

In early 2024, a multidisciplinary team of specialists was formed to control the outbreak. In this study, we aimed to resolve the outbreak through optimizing environmental sampling to guide cleaning and source investigation and by using genome sequencing on a broad panel of *A. flavus* from the outbreak, the hospital environment, and epidemiologically unrelated patients and sites. We also used time-scaled phylogeny to understand the evolution of the outbreak isolates. Although the comparison of typing methodologies is not a novel approach in a clinical context (*6*–*10*), this study also applied an extensive genomics approach with time-scaled phylogeny to an ongoing *Aspergillus* outbreak. Leveraging phylogenetics delineated the evolution of isolates within an *A. flavus* population that inhabited the hospital environment and successfully pinpointed a plausible source of contamination, as well as an unexpected timing of introduction.

## Materials and Methods

### Environmental Sampling and Comparison of Sampling Strategies

We took air samples (1 m^3^) using either a Microbio MB1 bioaerosol sampler (Cantium Scientific, https://www.cantiumscientific.com) onto 5 cm-diameter V8 agar contact plates (Bioneer A/S, https://bioneer.dk) or a MBASS30 V3 air sampler (Holbach, https://www.holbach.biz) onto 9-cm diameter V8 agar petri dishes (Bioneer A/S). We took surface samples using 5-cm diameter (25 cm^2^) V8 agar contact plates (Bioneer A/S), preferably from infrequently cleaned surfaces (*11*). We incubated 1 set of contact plates and petri dishes at 25°C and read after 7 days and incubated the other set at 37°C and read after 3 days. Air sampling was nonaggressive because disturbing dust was unacceptable for hospital staff.

**Figure 1:**
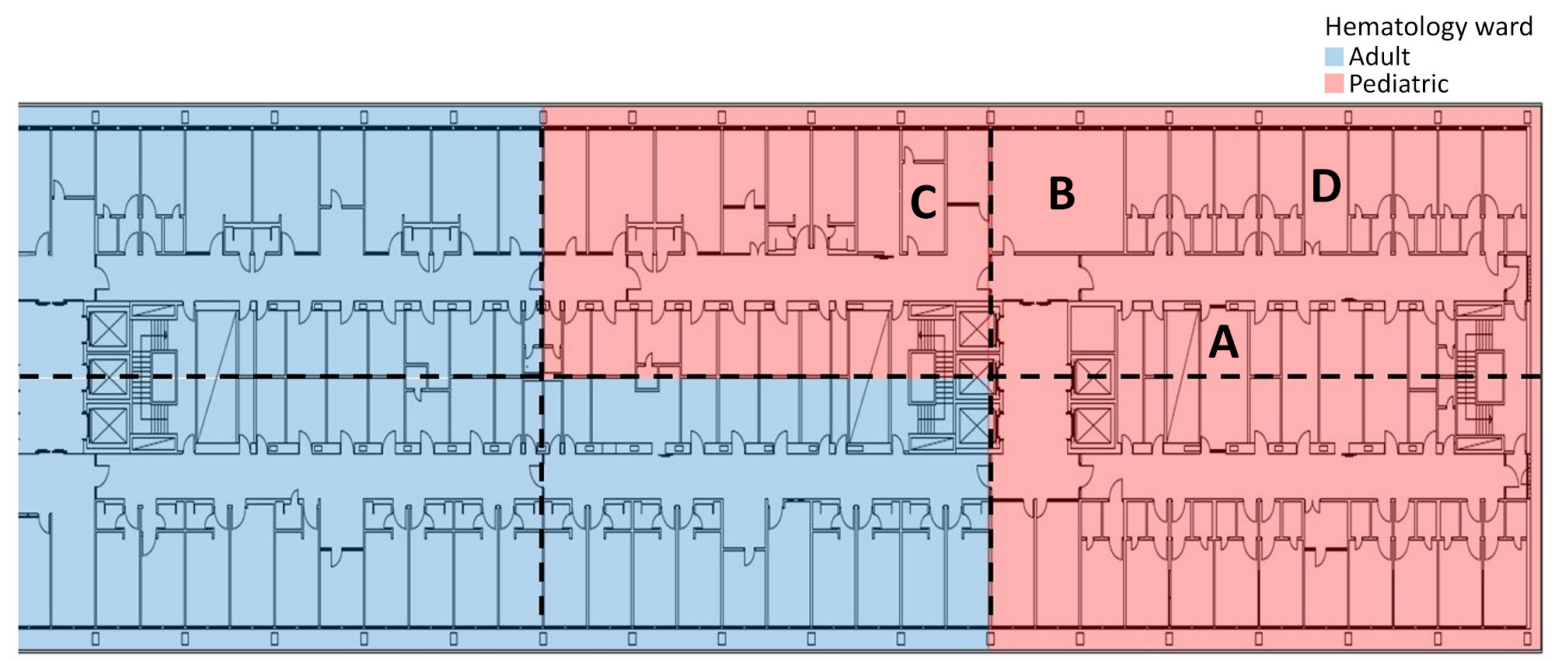
Floorplan of the outbreak floor in hospital South complex in study of environmental and phylogenetic investigations of *Aspergillus flavus* outbreak linked to contaminated building materials, Denmark, 2025. The hospital consists of 3 complexes: north, central, and south. The building is divided by corridors and staff rooms, with patient rooms facing the windows. Room A, staff meeting and lunchroom; room B, main kitchen; rooms C and D, staff offices. Figure created in BioRender (https://biorender.com/p56n130).

To assess *A. flavus* contamination and general fungal load, hospital sampling in March–June 2024 yielded 449 samples from 299 locations (in total). In March, we conducted environmental sampling at 41 predefined locations using V8 agar (*12*). At each location, we collected 4 samples (164 samples in total): 1 air sample incubated at 25 °C, 1 air sample incubated at 37 °C, 1 surface sample incubated at 25 °C, and 1 surface sample incubated at 37 °C. In addition, we investigated outdoor air controls and some sporadic surface samples (24 samples from 12 locations). Thus, in total, we collected 188 samples from 53 locations in the pediatric outbreak ward and outside the hospital. In April–June, we conducted another session focused on surface sampling and culturing at 37°C from the outbreak floor and collected 97 samples from 87 locations in both the pediatric and the adult hematology ward (Figure 1). That secondary sampling focused on areas that were most contaminated with *A. flavus* in the initial (paired) sampling session. Outside the outbreak floor, we took another 164 samples from 159 locations in high activity areas. We compared *A. flavus* CFU counts for paired samples in Rstudio using a χ^2^ test (using table 2×2 function, R version 4.2.3, and the package *Publish*) (The R Project for Statistical Computing, https://www.r-project.org).

We verified active growth of *A. flavus* on materials using tape preparations, microscopy (*12*), and matrix-assisted laser desorption/ionization time-of-flight mass spectrometry for identification (*13*). We collected soil samples (n = 8) from the hospital surroundings near the outbreak-affected South complex using a method described elsewhere (*14*) and cultured them at 37°C. Four *A. flavus* isolates were previously isolated from wood-based building materials, purchased from 2 retailers in Denmark, in an unrelated study of precontaminated materials (B. Andersen, unpub. data).

### STR*Afla* of Environmental Isolates

We applied STR*Afla* genotyping on 145 *A. flavus* isolates as previously described (*4*,*5*). Of those, 33 isolates (24 from patients and 9 from air) were included in our previous study (*5*). We genotyped additional patient isolates (n = 11), hospital interior isolates (n = 87 [1 isolate per positive location]), soil isolates (n = 10), and building material isolates (n = 4).

### Whole-Genome Sequencing of *A. flavus* Isolates

The dataset included 92 clinical and 17 environmental isolates from the outbreak hospital, 4 from unrelated wood-based materials, and 54 comparator isolates (167 isolates in total). All comparators, except 2, were previously STR*Afla* typed (*5*), and all originated either from hospital patients in Denmark who were not related to the outbreak or were control strains (4 UK-NEQAS and 2 ATCC 204304/200026 [CBS 128202] isolates).

We prepared sequencing libraries using a custom modified version of the DNA prep kit (Illumina, https://www.illumina.com) based on the Hackflex protocol (*15*) using 20 ng (2 ng/µL) genomic DNA as input and 25 µL of a 2× laboratory-made tagmentation buffer (20 mM Tris-HCl at pH 7.6), 20 mM MgCl2, and 10% (vol/vol) Propylene Carbonate (all Sigma-Aldrich, https://www.sigmaaldrich.com). We indexed the generated libraries with IDT for Illumina Unique Dual indexes and quantified them using the Quant-iT dsDNA HS Assay Kit (Thermo Fisher Scientific, https://www.thermofisher.com). We calculated molar concentrations using a standard conversion factor of 2 and pooled the samples at equimolar ratios. We assessed the quality of the final sequencing libraries by measuring concentration on a Qubit 3.0 fluorometer using the Qubit dsDNA HS quantification assay kit (Thermo Fisher Scientific) and by performing a fragment analysis using a TapeStation 4200 with the D5000 Screen Tape assay kit (Agilent, https://www.agilent.com). We sequenced quality checked final libraries on a NovaSeq (Illumina) using an S4 flowcell and 200-cycle reagent kit generating 2 × 100-bp paired-end reads.

We assessed quality and depth using NASP version 1.2.1 (*16*) and contamination screened using Kraken version 1.0 (*17*). We performed assembly with SPAdes version 3.11.1 (*18*) using the “–isolate” flag. We called single-nucleotide polymorphisms (SNPs) with NASP version 1.2.1 (*16*) aligned to NRRL 3357 (reference genome CBS128202, GenBank accession no. GCF_014117465) using BWA-MEM (H. Li, unpub. data, https://arxiv.org/abs/1303.3997v2) and GATK, requiring >10× depth and >90% unambiguous base calls. We removed recombination-prone regions using Gubbins version 2.3.4 (*19*) and inferred phylogenies with IQTREE version 2.0.3 (*20*) using the integrated model tester with ascertainment bias correction (-m TEST+ASC). Time-scaled phylogeny of cluster 10 used core-genome SNPs with strain T21212 as reference. We used BEAST2 (https://www.beast2.org) with sample dates as tips, BEAST Model Test, a relaxed clock model, and coalescent exponential population, with Markov chain Monte Carlo lengths of 200 million. We inferred clusters using TreeCluster (*21*) with the Max Clade method at a threshold of 0.01 for the full dataset and 0.001 for the cluster 10 tree. Furthermore, we inferred STR*Afla* locus 2A ancestral state in R version 4.4.2 using the ace function from the package *ape* under an equal-rates model.

## Results

### Environmental Sampling

**Figure 2:**
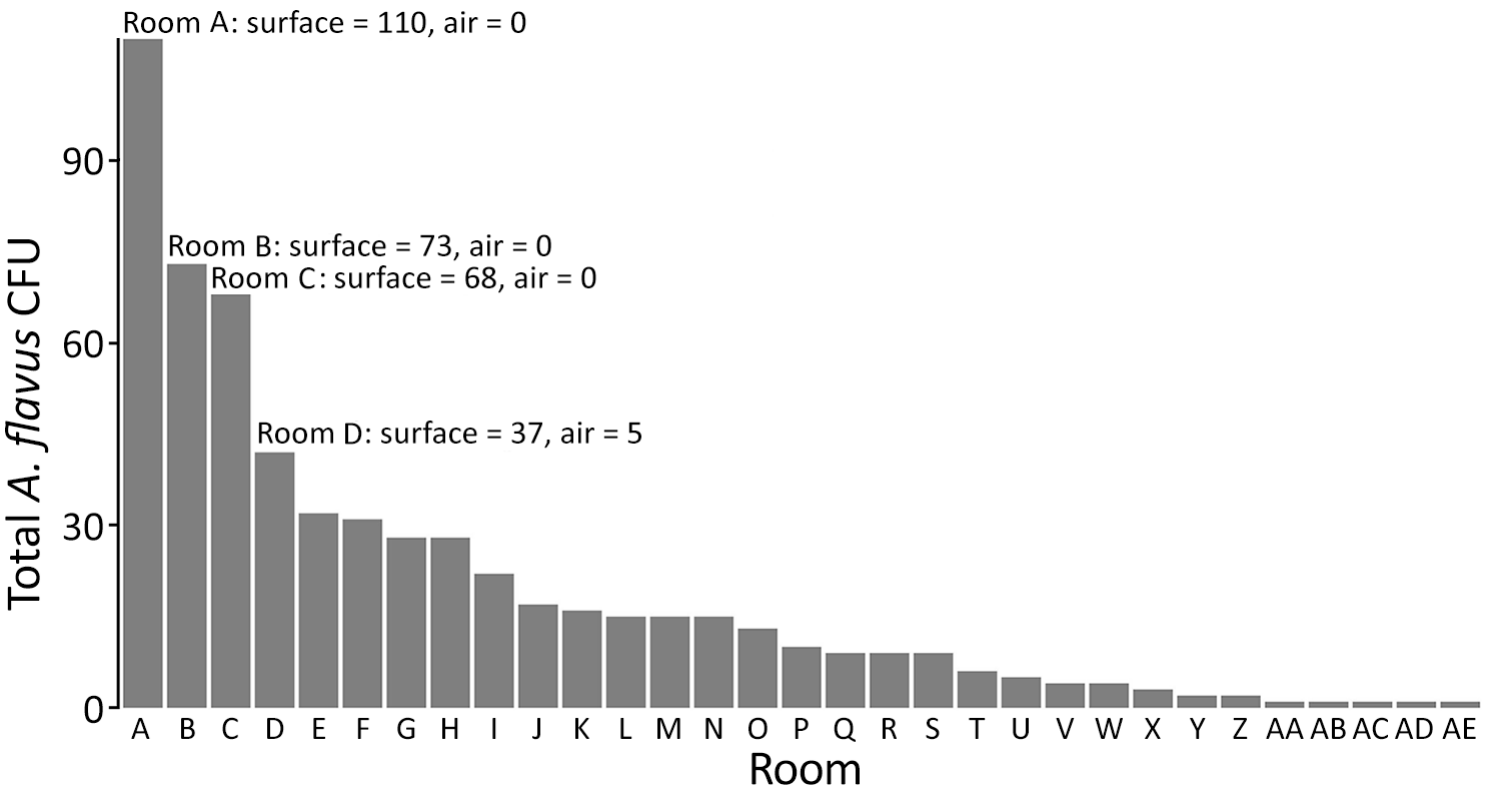
Distribution of *Aspergillus flavus* in hospital pediatric hemotology ward in study of environmental and phylogenetic investigations of *A. flavus* outbreak linked to contaminated building materials, Denmark, 2025. Bar chart displays total *A. flavus* CFU from paired samples in March 2024 (82 air samples and 82 surface samples incubated at 25°C or 37°C). Distribution in rooms of the ward shows varying concentration of spores with some hotspots of *A. flavus* contamination.

For initial sampling and comparison of strategies, paired sampling with culture on V8 agar yielded 1,712 fungal CFU, of which 775 were classical opportunistic species (including *Aspergillus flavus*, *A. fumigatus*, *A. niger*, and *Mucorales* spp.) and 593 were *A. flavus* specifically (Table 1; Figure 2). Surface sampling was more sensitive than air sampling overall (1,165 CFU [surface] vs. 547 CFU [air]), for the classical opportunistic species (731 CFU [surface] vs. 30 CFU [air]), and for *A. flavus* specifically (585 CFU [surface] vs. 8 CFU [air]) in the paired 81 air versus 81 surface samples. Culturing surface samples at 37°C increased *A. flavus* yield 1.77-fold (95% CI 1.57–1.99-fold; p<0.001). For air samples, the total CFU counts varied 9.2 fold at the 2 temperatures mainly because of notably fewer *A. calidoustus*, *Cladosporium*, and *Penicillium* spp. isolates when incubating at 37°C. Outdoor air controls grew *Cladosporium* and *Penicillium* spp. isolates but not *A. flavus* isolates.

**Table 1:** Comparison of fungal yield for paired sampling of air and surfaces in study of environmental and phylogenetic investigations of *Aspergillus flavus* outbreak linked to contaminated building materials, Denmark, 2025*

Identification	Air, n = 82						Surface, n = 82					Total CFUs
	25°C			37°C			25°C			37°C		
	CFU (CFU/m^3^)	Median (range)		CFU (CFU/m^3^)	Median (range)		CFU (CFU/25 cm^2^)	Median (range)		CFU (CFU/25 cm^2^)	Median (range)	
*Aspergillus*	186 (4.5)	0.0 (0–28)		26 (0.6)	0.0 (0–13)		321 (7.8)	5.0 (0–55)		491 (12.0)	6.0 (0–70)	1,024
*A. calidoustus*	179 (4.4)	0.0 (0–28)		3 (0.1)	0.0 (0–1)		13 (0.3)	0.0 (0–4)		39 (1.0)	0.0 (0–27)	234
***A. flavus***	**3 (0.1)**	**0.0 (0–1)**		**5 (0.1)**	**0.0 (0–4)**		**236 (5.8)**	**2.0 (0–55)**		**349 (8.5)†**	**2.0 (0–63)**	**593**
*A. fumigatus*	2 (0.0)	0.0 (0–1)		18 (0.4)	0.0 (0–13)		1 (0.0)			41 (1.0)	0.0 (0–14)	62
*A. niger*	2 (0.0)	0.0 (0–1)		0 (0.0)			42 (1.0)	0.0 (0–23)		62 (1.5)	0.0 (0–34)	106
*Aspergillus* NI	0 (0.0)			0 (0.0)			29 (0.7)	0.0 (0–25)		0 (0.0)		29
Other fungi												
*Alternaria*	4 (0.1)	0.0 (0–3)		0 (0.0)			38 (0.9)	0.0 (0–6)		1 (0.0)		43
*Aureobasidium*	6 (0.1)	0.0 (0–2)		0 (0.0)			6 (0.1)	0.0 (0–6)		2 (0.0)	0.0 (0–1)	14
*Cladosporium*	154 (3.8)	2.0 (0–23)		0 (0.0)			131 (3.2)	1.0 (0–15)		0 (0.0)		285
*Geotrichum*	0 (0.0)			1 (0.0)			0 (0.0)			0 (0.0)		1
*Mucorales*	2 (0.0)	0.0 (0–1)		2 (0.0)	0.0 (0–1)		8 (0.2)	0.0 (0–1)		2 (0.0)	0.0 (0–1)	14
*Penicillium*	118 (2.9)	2.0 (0–20)		26 (0.6)	0.0 (0–10)		135 (3.3)	0.0 (0–33)		24 (0.6)	0.0 (0–19)	303
Molds NI	20 (0.5)	0.0 (0–16)		0 (0.0)			1 (0.0)			3 (0.1)	0.0 (0–2)	24
Growth NI	2 (0.0)	0.0 (0–1)		0 (0.0)			0 (0.0)			2 (0.0)	0.0 (0–2)	4
No growth	3			26			3			13		45
Fungi in total	492 (12.0)	9.0 (0–44)		55 (1.3)	0.0 (0–13)		640 (15.6)	13.0 (0–59)		525 (12.8)	6.0 (0–71)	1,712

*Samples were taken from 41 locations on the outbreak floor in March 2024 and cultured at 25°C or 37°C . Bolding highlights *A. flavus* findings. NI, not identified. †p<0.001 for culturing surface samples at 37°C versus 25°C (*A. flavus*).

**Figure 3:**
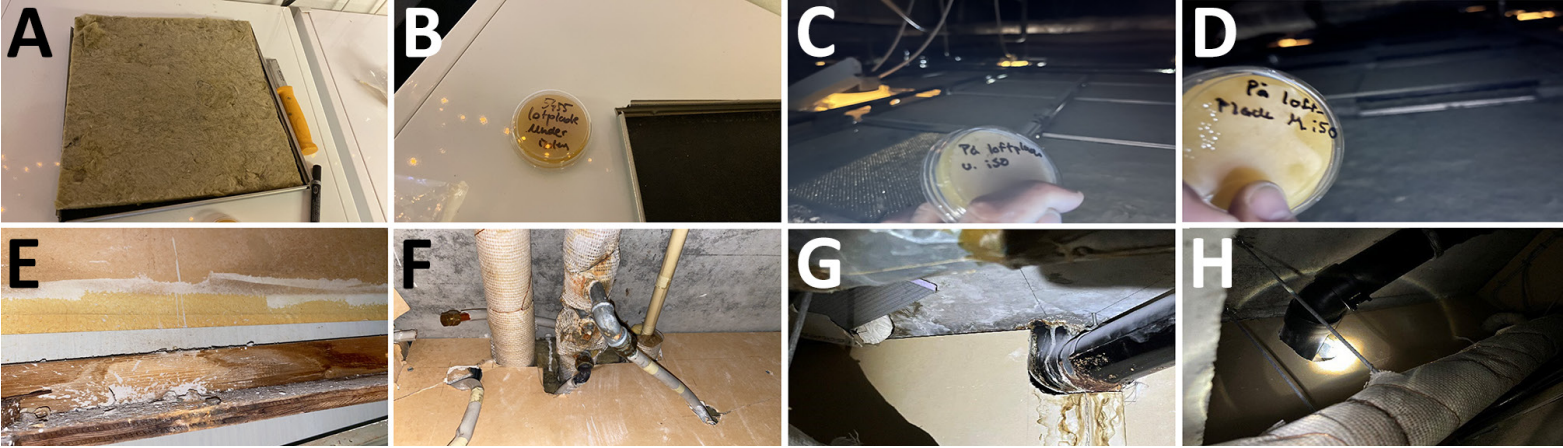
Examples of locations with dust accumulation above ceiling and large *Aspergillus flavus* contamination from hospital pediatric hemotology ward in study of environmental and phylogenetic investigations of *A. flavus* outbreak linked to contaminated building materials, Denmark, 2025. A) Ceiling tile from room A with 55 CFU/25 cm^2^ *A. flavus* detected on top (none detected underneath); B) the same ceiling tile (black item) from room A with the insulation sheet (yellow mat) removed, where the surface had been covered and thus tested negative for *A. flavus*; C) area above ceiling in room B, 24 CFU/25 cm^2^ (no rock wool); D) thick dust layer on inside of ceiling, room B; E) example of a water-damaged part of the wooden framing with mold-like growth above the suspended ceiling, 32 CFU/25 cm^2^; F) 40 CFU/25 cm^2^ on righthand pipe; G, H) pipes between rooms lacking sealing, which could possibly enable spore dispersal.

We performed subsequent sampling during April–June 2024 with incubation at 37°C in 15 pediatric ward locations, which yielded a total of 324 *A. flavus* CFU (median 13.0 [range 0–58] CFU/25 cm^2^). Additional sampling above the ceiling gave 195 *A. flavus* CFU (median 4.0 [range 0–55] CFU/25 cm^2^). Samples beneath insulation sheets on the ceiling tiles were negative (Figure 3). In the adjacent adult hematology ward (Figure 1), we found 527 *A. flavus* CFU (median 1.0 [range 0–53] CFU/25 cm^2^). On 10 other floors, we collected 164 surface samples; we found *A. flavus* in 5 samples (33 CFU total); 29 were from a kitchen refrigerator top (central complex) and 4 were scattered elsewhere.

**Figure 4:**
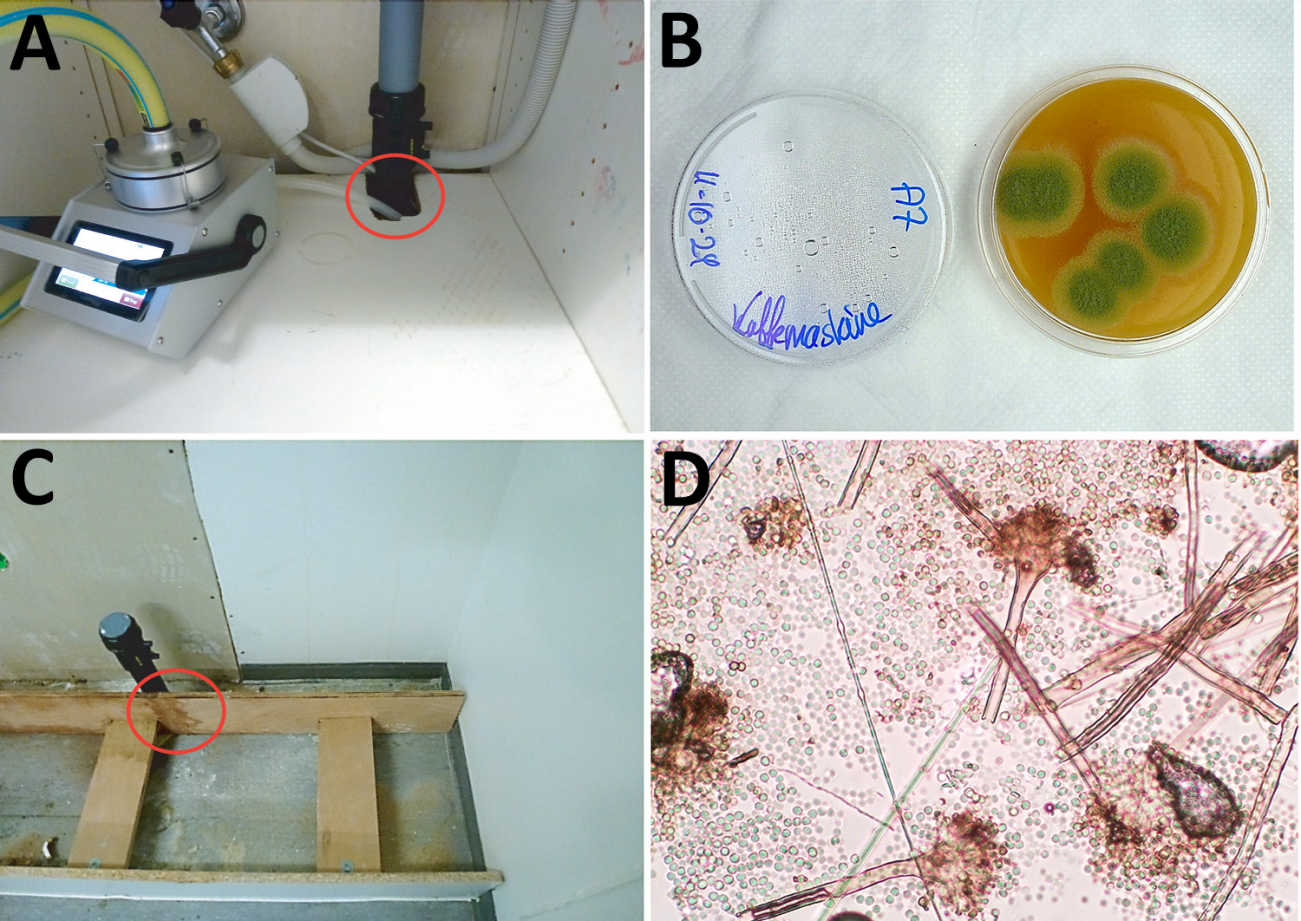
Testing materials from room A in hospital pediatric hemotology ward in study of environmental and phylogenetic investigations of *A. flavus* outbreak linked to contaminated building materials, Denmark, 2025. A) Directed air sampling under the kicthenette with a polyethylene tube (red circle). B) V8 contact plate from the top of the coffee machine showing *A. flavus* colonies after 3 days’ incubation at 37°C. C) Discolored plywood (red circle) next to the drain after dismantling the cupboard. D) Tape preparation of the discolored area showing *A. flavus* stipes, heads, and spores.

Peak *A. flavus* contamination was found in a combined staff meeting and lunchroom (room A), the main ward kitchen (room B), and staff offices (rooms C–D) (Figures 1–3), especially on less frequently cleaned surfaces. In November 2024, directed air samples suggested that the kitchenette base under the sink in room A was a source of contamination; yield was 1,500 CFU/m^3^. After dismantling, microscopy confirmed growth of *A. flavus* on a plywood cupboard (Figure 4).

### STR*Afla* of Environmental Isolates

We genotyped a total of 145 *A. flavus* isolates using STR*Afla*, revealing 28 unique genotypes (Table 2). The dominant outbreak genotype, A, was identified in 19 isolates from 11 patients and 82/96 indoor hospital isolates and occurred exclusively on the outbreak floor, in both the adult and pediatric hematology wards (Table 2; Figure 1). In the adult ward specifically (Figure 1), 36/38 isolates shared genotype A, whereas 2 were different (Table 2). Eight isolates from 7 patients and 5 isolates from a contaminated DCM incubator shared genotype B, in which 1 of 9 markers (marker 2A) differed from that of genotype A by 1 repeat. Eight additional patient isolates shared 7–8 markers; genotype A displayed different marker variations across the STR*Afla* panel (Table 2). Among the 82 hospital interior isolates with genotype A, 2 were from the water-damaged plywood cupboard in room A (Table 2; Figure 4). STR*Afla* of 4 outbreak-unrelated *A. flavus* isolates from wood-based panels (medium-density fiberboard, oriented strand board, and plywood) obtained from 2 retailers in Denmark and cultured outside this study, revealed partial similarity to genotype A with 5–8 of 9 shared markers (genotypes C, D, E) (Table 2). Remaining genotypes differed from the outbreak genotype A by >5 markers, as did soil isolates (Table 2).

**Table 2:** *Aspergillus flavus* genotypes found in 145 isolates in study of environmental and phylogenetic investigations of *Aspergillus flavus* outbreak linked to contaminated building materials, Denmark, 2025*

Source of isolate(s) (genotype)	No. isolates	STRA*fla*-markers									Comments
		2A	2B	2C	3A	3B	3C	4A	4B	4C	
Patients 1, 2, 4, 6, **9**, 10, 16, 17, 18, 20, 21 (genotype A)†	19	26	11	12	12	26	9	9	5	9	
Patient 21	3	26	11	12	12	25	9	9	5	9	
Patients **5**, 7 ,**12–14**, 15, 19 (genotype B)	8	25	11	12	12	26	9	9	5	9	
Patient 11	1	26	11	12	12	27	9	9	5	9	
Patient 11	1	27	11	12	12	27	9	9	5	9	
Patient **8**	1	26	11	14	12	26	9	9	5	9	
Patient 3	1	25	11	12	12	26	9	9	5	NA‡	
Patient 15	1	25	11	12	12	26	13	9	5	9	
Air samples (genotype A)†	9	26	11	12	12	26	9	9	5	9	Pediatric hematology ward
Plywood cupboard in room A§ (genotype A)†	2	26	11	12	12	26	9	9	5	9	Pediatric hematology ward
Vacuum cleaner bag (genotype A)†	2	26	11	12	12	26	9	9	5	9	Pediatric hematology ward
DCM laboratory incubator (genotype B)	5	25	11	12	12	26	9	9	5	9	
Hospital interior (genotype A)†	69	26	11	12	12	26	9	9	5	9	Adult/pediatric hematology ward
Hospital interior	1	62	11	12	9	20	9	7	12	10	Central complex
Hospital interior	1	40	12	11	8	13	4	7	7	9	South complex¶
Hospital interior	1	31	11	10	8	13	14	7	12	10	Adult hematology ward
Hospital interior	2	27	13	8	14	29	9	9	5	9	Central complex
Hospital interior	1	20	16	11	10	22	32	7	8	8	Pediatric hematology ward
Hospital interior	2	15	16	28	11	17	15	7	8	9	South complex¶
Hospital interior	1	14	11	10	8	22	9	7	12	10	Adult hematology ward
MDF (genotype C)	1	26	11	13	12	28	9	9	5	9	Retailer 1
MDF (genotype D)	1	NA‡	NA‡	NA‡	12	27	9	9	5	9	Retailer 2
OSB (genotype E)	1	26	11	12	12	27	9	9	5	9	Retailer 1
PW (genotype E)	1	26	11	12	12	27	9	9	5	9	Retailer 1
Soil-1a	1	15	7	22	16	12	16	9	5	8	
Soil-1b	1	15	7	23	17	13	5	9	5	8	
Soil-6a	1	15	11	12	8	30	14	8	7	10	
Soil-7a	1	16	7	11	19	6	5	7	5	8	
Soil-7b	1	30	11	11	8	24	15	7	7	10	
Soil-7c	1	15	7	21	18	12	11	10	5	8	
Soil-7d	1	28	11	11	8	20	9	7	12	10	
Soil-7e	1	29	11	11	8	23	15	7	7	10	
Soil-7f	1	16	7	33	14	6	11	17	5	8	
Soil-7g	1	39	11	10	8	45	9	7	12	10	
Total	145										

*Including 33 isolates compiled from Gewecke et al. (*6*). Patient numbers in bold indicate cases in which culture-plate contamination was assumed because of lack of symptoms, lack of statements in the medical records, or both. Gray shading indicates STRA*fla* marker overlaps with outbreak. MDF, medium-density fiberboard; OSB, oriented strand board; PW, plywood. †Principal South complex outbreak cluster genotype A.  ‡No STRA*fla* profile.  §Microscopy-confirmed growth of *A. flavus* on kitchen plywood cupboard in room A (see Figure 4). ¶South complex but different floor than outbreak ward.

### Whole-Genome Sequencing–Based Population Analysis of *A. flavus* Isolates

**Figure 5:**
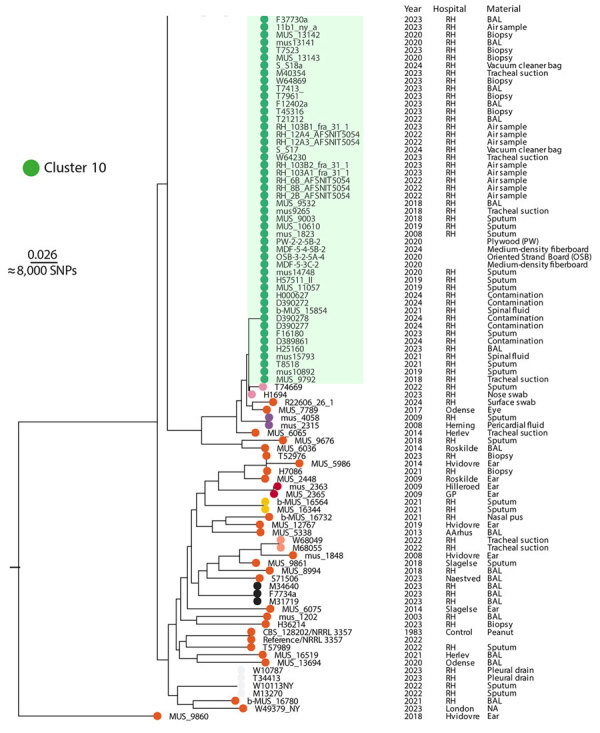
Rooted maximum-likelihood phylogeny of 167 *A. flavus* isolates (cropped) from study of environmental and phylogenetic investigations of *A. flavus* outbreak linked to contaminated building materials, Denmark, 2025. The tree shows clear distinction of several major clusters, including 1 monophyletic cluster 10 that contained all outbreak isolates. The tree was reconstructed based on 303,560 core-genome SNPs and rooted with MUS_9860 as outgroup. Scale bar indicates substitutions per site. Clusters were determined using TreeCluster (https://github.com/niemasd/TreeCluster). Full tree available in Appendix 1. BAL, bronchoalveolar lavage; NA, not available (information on material source); RH, Rigshospitalet; SNP, single-nucleotide polymorphism.

A total of 167 isolates underwent whole-genome sequencing. We aligned reads to the reference genome and identified core-genome SNPs. After excluding recombination regions, we retained 303,560 SNPs in the reference chromosome for phylogenetic inference. This process produced a structured tree with distinct clusters separated by thousands of SNPs (Figure 5). Outbreak isolates grouped into cluster 10, which distinctly separated from others and showed minimal within-cluster variation indicating high genetic similarity. All cluster 10 isolates originated from the hospital except for 4 building material isolates.

**Figure 6:**
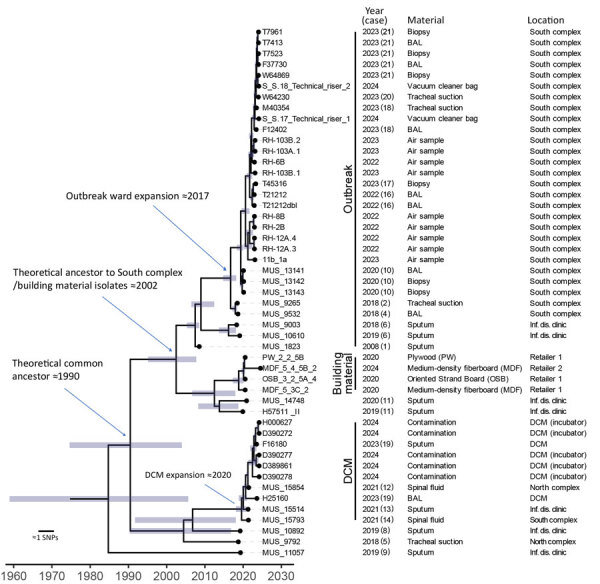
Time-scaled core-genome SNP-based phylogeny of cluster 10, which contained all outbreak isolates, in study of environmental and phylogenetic investigations of *A. flavus* outbreak linked to contaminated building materials, Denmark, 2025. Bars around internal nodes indicate the 95% highest posterior density (HPD) interval of the branching time. There is a distinct clustering of the South complex outbreak isolates (indicated as Outbreak), the isolates from building materials, and isolates from the DCM versus other quality control strains or strains from Denmark (Figure 4). Dating of ancestral nodes indicates that a common ancestor to the outbreak floor clonal expansion existed in the South complex around 2017 (95% HPD 2015–2018). The isolates from the outbreak floor are closely related with the building material isolates and share an inferred ancestor in 2002 (95% HPD 1995–2008). An expansion of a more recent ancestor occurred in the DCM around 2020 (95% HPD 2018–2021), detected as contamination in a laboratory incubator entailing an infection in >1 patient (case 19) and possibly also contamination of clinical samples (case 12, 13, 14) based on medical records. Earliest inferred ancestor to all outbreak isolates possibly existed around 1990 (95% HPD 1975–2004). The consensus tree was obtained using BEAST2 (https://www.beast2.org) on 723 core-genome SNPs called using an internal reference (T21212). *A. flavus* from patients 3, 7, and 15 were not available for sequencing. BAL, bronchoalveolar lavage; DCM, Department of Clinical Microbiology; inf. dis., infectious disease; SNP, single-nucleotide polymorphism.

Using an internal outbreak draft genome as reference, resolution increased and enabled further analyses. In the cluster-specific tree (Figure 6), we retained 723 SNPs for time-scaled phylogenetic reconstruction. South complex and building material isolates were close, but the building material isolates clustered more basal alongside 2 isolates from patient 11 (infectious disease clinic). The last common ancestor of South complex and building material isolates likely dates to 2002 (95% highest posterior density [HPD] 1995–2008). South complex expansion occurred around 2017 (95% HPD 2015–2018). Isolates were distinct from the DCM isolates, which expanded around 2020 (95% HPD 2018–2021). A common ancestor to that monophyletic cluster dates back to ≈1990 (95% HPD 1975–2004).

Infectious disease clinic isolates were mainly from outpatients and considered noninfected. Those isolates displayed different STR*Afla* variants (Figure 6). Patients 6 and 11 were colonized and culture-positive twice. In both cases, the patients retained their original genotypes over a 1-year period. Isolates from patients 5, 8, 9, and 12–14 were regarded as plate contaminants because of growth outside the inoculation zone, lack of clinical signs, or both. European Organisation for Research and Treatment of Cancer classification (into proven, probable, possible, or no infection) remains unverified (*22*).

Phylogenetic analysis also showed that STR*Afla* marker 2A was able to consistently differentiate South complex from DCM isolates (26 repeats for South complex vs. 25 repeats for DCM isolates) (Table 2). The ancestral genotype of both were most likely genotype A with 26 repeats on the basis of an inferred ancestral state.

## Discussion

This study highlights some of the challenges faced in an *Aspergillus* outbreak investigation, the importance of optimal sampling and identification strategies, and the valuable insights provided by modern typing and whole-genome sequencing techniques. Environmental sampling was challenging. Air sampling notably underestimated the overall burden of *A. flavus* on the outbreak floor, whereas in this investigation surface sampling proved notably more sensitive, not only for *A. flavus* but also for *A. fumigatus* and *A. niger*. Surface sampling established a contamination gradient across the outbreak floor (Figures 2, 3) and thus enabled the discovery of a plywood cupboard with microscopy-confirmed *A. flavus* growth (room A) (Figure 4). Phylogeny revealed a close link between the outbreak isolates and 4 *A. flavus* isolates from unused wood-based building materials sourced from 4 retailers in Denmark (Figure 6). Alongside confirmed in situ growth on 1 such material inside the ward (Figure 4), those findings suggest precontaminated building materials as a potential origin of the outbreak. On the basis of 95% HPD interval, a common ancestor to cluster 10 might have entered during initial hospital construction (≈1970s) or during later renovations. As previously speculated, the South complex isolates likely spread during ward renovations during 2017–2019 (*5*), coinciding with the outbreak onset, in accordance with this study’s detection of clonal expansion in the South complex occurring at that time (Figure 6).

Consensus guidelines regarding how to perform and interpret *Aspergillus* hospital outbreak investigations are currently sparse and linked to air sampling. Ruiz-Camps et al. (*23*) recommended a limit of 0.5 fungal CFU/m^3^ in HEPA-protected air and 25 CFU/m^3^ in unprotected air but did not specify a culture temperature. Chang et al. (*24*) suggested limits of <5 *Aspergillus* CFU/m^3^ in protective isolation areas and <0.1 *Aspergillus* CFU/m^3^ in HEPA-filtered environments, along with a threshold of 15 fungal CFU/m^3^ for gross colony counts. Moreover, culture at 37°C was recommended to favor growth of human pathogenic fungi (*24*). However, Morris et al. (*25*), using the same thresholds, advised culturing at 28°C. Those discrepancies are not trivial and may result in divergent risk assessment. Optimal growth conditions for *A. flavus* are 32°C–33°C, substrate water activity of 0.95, and pH 4–6.5 (*26*,*27*). However, it grows well at 37°C (*28*) and can grow at 20°C–40°C, substrate water activity of >0.80, and pH 4–9 (*26*,*27*). In addition, *A. flavus* can grow in a variety of substrates because of its abundant enzyme production (*11*). In our study, mean total fungal CFU counts varied 9-fold for paired air samples cultured at the 2 temperatures, mainly because of *A. calidoustus*, *Cladosporium*, and *Penicillium*, which dominated spore counts at 25°C but rarely cause infections. Perhaps even more concerning, air sampling was overall notably insensitive to detect *A. flavus*. Thus, surface sampling from infrequently cleaned sites is key for detection; this sampling substantially increased the yield of *A. flavus* (as well as of *A. fumigatus* and *A. niger*), in agreement with previous observations (*11*,*29*). Moreover, this study suggests incubation at 37°C to favor *A. flavus* (and other opportunistic molds) and to minimize overgrowth of irrelevant species, thereby avoiding difficulties in plate reading and underestimation of the *A. flavus* burden (Table 1). Finally, the dispersal of spores throughout the outbreak floor indicates that settled dust in less frequently cleaned areas might serve as satellite sources of spores with dust-mediated spread of *A. flavus* (*30*).

STR*Afla* typing identified the outbreak genotype A exclusively on the outbreak floor (Table 2) during the environmental sampling session March–June 2024. Closely related isolates came from 7 additional patients, a DCM incubator (5 isolates) (shared 8/9 markers, genotype B), and from wood-based materials (4 isolates) that shared 5–8/9 (genotypes C, D, E) STR*Afla* markers with the outbreak genotype A. Of note, individual STR*Af* markers in *A. fumigatus* might gain single-repeat variants over serial propagation in vivo and in vitro as a result of microevolution (*31*); thus, we initially regarded genotype A and B as identical and equally involved in the outbreak despite a 1-repeat difference (Table 2). However, genome-based phylogenetic analysis confirmed that degrees of isogeneity among *A. flavus,* observed through STR*Afla* typing were consistent. Single-repeat differences at 2A are likely to be genetically meaningful, and provided laboratories continuously use matching protocol parameters, interpretability in regards to single-repeat deviances should not be a concern (*32*). The relatively small variations in repeat counts in the genotypes of wood-based isolates might also reflect the evolutionary biology of STR sequences that possibly evolve through small, stepwise repeat changes over time (*33*,*34*). Taken together, our results support STR*Afla*’s continued value as a sensitive and specific frontline screening tool for clinical outbreak investigations. Furthermore, those findings are consistent with those of other studies validating microsatellite assays in fungi through genome sequencing (*7*,*9*,*10*), although species-dependent variation in discriminatory power might exist.

Core-genome phylogeny revealed clusters across the isolate collection; cluster 10 (Figures 5, 6) distinctly separated from other clusters. Cluster 10 isolates only included isolates from the outbreak hospital, except those from building materials, supporting a localized outbreak. Minimal genetic diversity within cluster 10 indicated recent clonal expansion.

Bayesian time-scaled phylogeny identified 3 cluster 10 genetic variants: 1 in the South complex, 1 in external building materials (and patient 11 [Figure 6]), and 1 in the DCM. The variants were detected at different time points, but a common ancestor could date back to the 1970s, coinciding with the construction of the hospital (Figure 6).

Confirmed growth of the South complex variant on a plywood base in the outbreak ward suggests a link to precontaminated wood-based materials, also observed for other molds (*35*). The plywood was part of a kitchen installed in 2012, raising 2 possibilities: that periodic hospital renovations and occasional water damage promoted growth of already prevalent spores that persisted and evolved within the hospital environment, or that repeated introductions to the hospital occurred, possibly through precontaminated wood-based materials. The second is supported by almost genetically identical *A. flavus* isolates found in hospital-unrelated wood-based materials from retailers in Denmark, but that theory does not explain the finding of an outbreak isolate in a patient sample as early as 2008. If precontaminated materials were the original source, dormant *A. flavus* spores might have germinated after water damage (*36*). Wood-based materials can act as *Aspergillus* substrates (*11*), and strains phylogenetically related to the outbreak isolates grew on both plywood and oriented strand board (Table 2; Figure 4). Whether cluster 10 represents a genetically distinct subdivision of *A. flavus* or whether genome rearrangements (*37*), other alterations (*38*), or selective pressures (*7*,*38*) drove its hospital expansion remains unresolved. However, our findings raise concern related to the use of wood-based materials (and organic building materials in general) in hospital areas housing immunocompromised patients.

In conclusion, this study underscores the importance of comprehensive and correct environmental sampling and genomic analysis in *A. flavus* outbreaks. Our understanding of this outbreak was substantially enhanced by the use of phylogenetic analysis. Dormant spores of *A. flavus* had begun actively growing within the hospital environment, with settled dust acting as a reservoir for conidia. Phylogeny validated STR*Afla* typing as a sensitive and specific tool for frontline outbreak investigation. Crucially, time-scaled phylogeny linked the outbreak isolates to *A. flavus* isolated from colonized wood-based materials, implicating the hospital’s original construction or subsequent renovations as the likely source of introduction. Our findings should be taken into consideration in planning for the material composition of future hospitals.

Appendix 1 Additional information about environmental and phylogenetic investigations of *Aspergillus flavus* outbreak linked to contaminated building materials, Denmark, 2025

Appendix 2 Additional information from study of environmental and phylogenetic investigations of *Aspergillus flavus* outbreak linked to contaminated building materials, Denmark, 2025
