# Supplementary material for: Environmental and Phylogenetic Investigations of Aspergillus flavus Outbreak Linked to Contaminated Building Materials, Denmark, 2025
Source: Emerg Infect Dis. 2026 Mar;32(3):376–87. doi: 10.3201/eid3203.251219 (PMC13016004; doi:10.3201/eid3203.251219)
Supplement: Appendix 1 — Additional information about environmental and phylogenetic investigations of Aspergillus flavus outbreak linked to contaminated building materials, Denmark, 2025 [file 25-1219-Techapp-s1.pdf]

Article DOI: https://doi.org/10.3201/eid3203.251219

EID cannot ensure accessibility for supplementary materials supplied by authors. Readers who have difficulty accessing supplementary content should contact the authors for assistance.

Environmental and Phylogenetic Investigations of *Aspergillus flavus* Outbreak Linked to Contaminated Building Materials, Denmark, 2025

Appendix 1

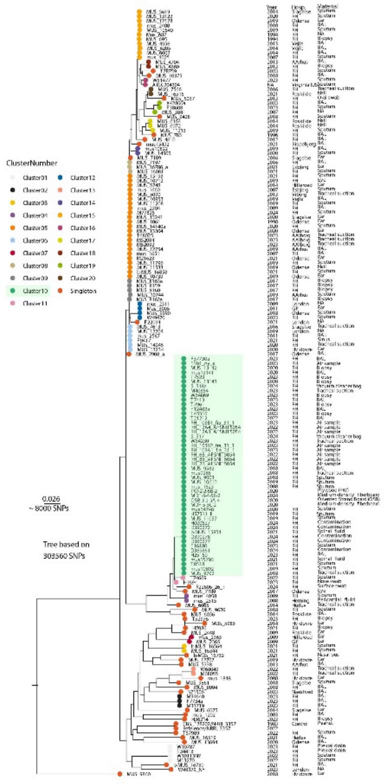

Appendix 1 Figure. Rooted maximum likelihood phylogeny of 167 A. flavus isolates. The tree shows clear distinction of several major clusters, including one monophyletic "Cluster 10" that contained all outbreak isolates. Sampling "Year," hospital "Hosp," and "Material" are indicated on the right-hand side of the corresponding isolate names. The tree was reconstructed based on 303,560 core-genome SNPs and rooted with MUS_9860 as outgroup. The scale bar indicates substitutions per site. Clusters were determined using TreeCluster.
